# Unveiling the high-temperature dielectric response of $\text {Bi}_{0.5}\text {Na}_{0.5}\text {TiO}_{3}$

**DOI:** 10.1038/s41598-020-75859-z

**Published:** 2020-11-10

**Authors:** Julio Cesar Camilo Albornoz Diaz, Jean-Claude M’Peko, Michel Venet, Paulo Sergio da Silva

**Affiliations:** 1 Department of Physics, Federal University of São Carlos, P.O. Box 676, São Carlos, SP 13565-905 Brazil; 2 Center of Science and Technology of Materials, Energy and Nuclear Research Institute, São Paulo, SP 05508-170 Brazil; 3 São Carlos Institute of Physics, University of São Paulo, P.O. Box 369, São Carlos, SP 13560-970 Brazil

**Keywords:** Condensed-matter physics, Ferroelectrics and multiferroics, Physics, Condensed-matter physics, Ferroelectrics and multiferroics, Materials science, Materials for devices, Actuators, Electronic devices

## Abstract

Understanding the physics behind changes in dielectric permittivity and mechanical response with temperature and frequency in lead-free ferroic materials is a fundamental key to achieve optimal properties and to guarantee good performance in the technological applications envisaged. In this work, dense $\text {Bi}_{0.5}\text {Na}_{0.5}\text {TiO}_{3}$ (BNT) electroceramics were prepared through solid-state reaction of high-grade oxide reagents, followed by sintering at high temperature (1393 K for 3 h). In good agreement with previous reports in the literature, the thermal behaviour of dielectric response from these BNT materials showed the occurrence of a high-temperature diffuse-like permittivity peak, whose origin has been so far controversial. Thermally stimulated depolarization current, impedance and mechanical spectroscopies measurements were here conducted, over a wide range of temperature and frequency, to get a deep insight into the mechanism behind of this event. The approach included considering both as-sintered and reduced BNT samples, from which it is demonstrated that the broad high-temperature dielectric peak originates from interfacial polarization involving oxygen vacancies-related space-charge effects that develop at the grain-to-grain contacts. This mechanism, that contributes to the anomalous behavior observed in the mechanical response at low frequencies, could also be responsible for the presence of ferroelastic domains up to high temperatures.

## Introduction

Lead zirconium titanate (PZT)-based piezoceramics are used in several commercial electro-electronic devices to accomplish varied functions, like step-down multilayer piezoelectric transformers for AC–DC converter applications, high energy capacitors, non-volatile memories (FRAM), ultrasonic sensors, infrared detectors, and electro-optic devices^1–3^. In terms of environmentally friendly systems, nevertheless, the search for lead-free alternative materials has grown quickly over the last decades^4–7^. In particular, $\text {Bi}_{0.5}\text {Na}_{0.5}\text {TiO}_{3}$ (BNT)-based materials have attracted great attention of scientists as a possible alternative to PZT-based piezoceramics^8–17^.

By using Neutron diffraction, Jones and Thomas^18^ reported that BNT presents a somewhat complex phase transition sequence on heating (from 5 K), consisting in rhombohedral (*R*3*c*) to tetragonal (*P*4*bm*) transition at 528 K, and then tetragonal to cubic ($Pm\overline{3}m$) at 813 K, with region of phases coexistence of rhombohedral plus tetragonal towards the 528–673 K range, and tetragonal plus cubic towards the 773–813 K range. By using transmission electron microscopy, on the other hand, Dorcet et al. observed a local modulated phase between 470 and 550 K, constituted of *Pnma* orthorhombic sheets between two *R*3*c* ferroelectric domains^19,20^. This rhombohedral/orthorhombic coexistence was also observed at room temperature by Jones et al.^21^ when pressure was applied to BNT crystals. According to the authors^19,20^, the modulated phase transforms to orthorhombic *Pnma* close to 550 K and persist up to approximately 590 K, where a phase transition to a globally disordered tetragonal structure (*P*4/*mbm*) with locally ordered ($P4_{2}/mnm$) nanodomains, occurs. However, this additional structural phase transition at around 590 K, or even the presence of the orthorhombic phase, is somewhat controversial. By using anelastic characterizations, which is an extremely sensitive technique to structural phase transitions, Cordero et al.^22^ have not observed evidence of any structural phase transition between 570 and 820 K. Indeed, the thermal evolution of the crystallographic structure in BNT, as proposed by Jones and Thomas^18^, has been generally adopted^22–24^.

In parallel, the temperature dependence of dielectric permittivity of BNT has been shown to exhibit a dielectric peak usually found to occur around $600 \le T_{m} \le 650 \,\text {K}$, where there exists however no evidence of structural transformation taking place, albeit report of some phases coexistence^18^, as commented above. This peak normally is extremely broad, extending to temperatures that may reach 800 K^22,24–28^, that is, approaching the temperature region of tetragonal to cubic phase transition^18,29^. This has allowed several authors^21,24,26,30–33^ to classify such a dielectric peak as representing a diffuse phase transition (DPT), but the origin of this supposed diffusivity remains controversial, being attributed in some cases to the reorientation of polar nano regions^20,26,34,35^, heterophase fluctuations^31,32^, or defect dipoles originated by oxygen vacancies^30^. Besides, Liu et al. have deconvoluted this broad high-temperature dielectric peak in BNT-based materials by using two Gaussian functions, that were associated with the rhombohedral *R*3*c* to tetragonal *P*4*bm* phase transition and with the clamping of domain walls into the polar regions, induced by oxygen vacancies^36^.

In last years, the analysis of the elastic constants and energy dissipation spectra of electroceramics materials by mechanical spectroscopy has been providing clear information about the occurrence and characteristics of phase transitions^16,22,37–41^, especially in materials with ferroelastic nature like BNT, relaxation mechanisms^42–44^ and defects mobility^44–46^. Nevertheless, only a few works in the literature address the use of this technique to investigate the phase transitions and other thermally stimulated processes in BNT-based materials^11,22,24,47–49^.

Here we present a detailed study based on thermally stimulated depolarization current (TSDC), impedance spectroscopy and mechanical spectroscopy approaches that allow demonstrating that the origin of the dielectric behaviour observed in BNT ceramics around $T_{m}$ is closely related with oxygen vacancies-associated space-charge effects developing at the grain-to-grain contacts with a clear contribution to the broadening of mechanical losses for temperatures between 600 and 800 K, in low frequencies.

## Results and discussions

### Dielectric, mechanical and TSDC characterizations

**Figure 1:**
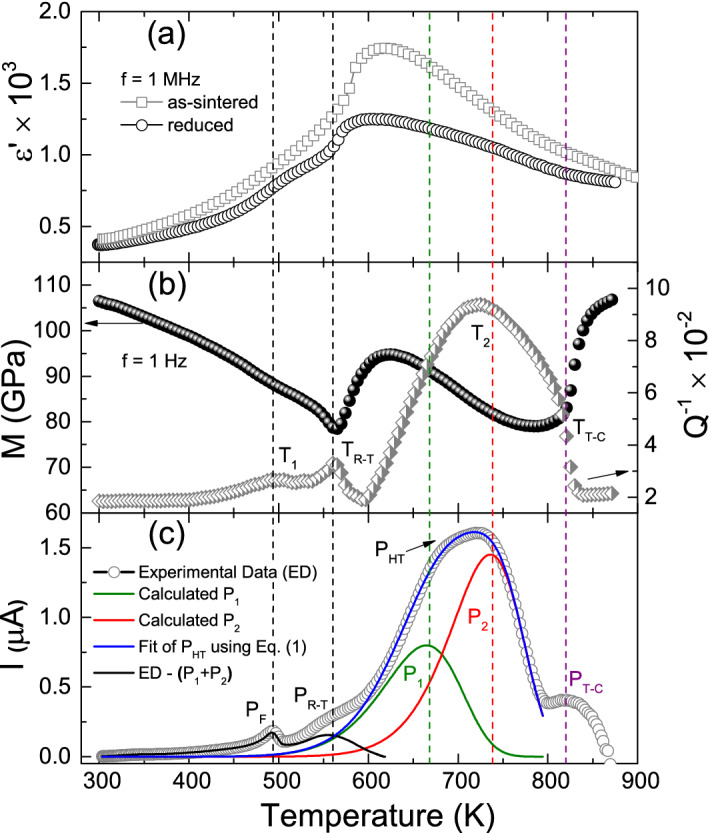
(**a**) Thermal behaviour of real part of permittivity for as-sintered and reduced BNT samples, (**b**) The Young’s modulus (M) and internal friction ($Q^{-1}$) as a function of temperature for as-sintered BNT at 1 Hz and (**c**) TSDC data from an as-sintered BNT sample measured from RT to 800 K, after a polarization process at 330 K under an electric field of 4 kV/mm, applied during 40 min; the observed peaks were labelled as $\text {P}_{F}$, $\text {P}_{R-T}$ and $\text {P}_{HT}$. Fit of the broad $\text {P}_{HT}$ peak (consisting of peaks $\text {P}_{1}$ and $\text {P}_{2}$) was conducted using Eq. (1) and the initial rise method (see text).

Figure 1a shows the temperature dependence of dielectric permittivity from both as-sintered and reduced BNT samples. Each curve shows a shoulder around 500 K, towards which ferroelectric depolarization occurs in BNT^19,21,22,30^. In the following, a broad dielectric peak develops around 610 K, where, as we earlier commented, no structural phase transformation has been found^18^. This anomaly, also reported elsewhere^22,24–28^, is the dielectric response of interest in this report. Because of its broadness, this peak has been connected to a diffuse-like phase transition by some authors^21,24,26,30–33,50^. Interesting is the fact that no important dispersion of this peak with frequency is normally found^25,51^, which excludes possibility of a relaxor-type phase transition.

Indeed, the above observations suggest that the origin of this dielectric peak in BNT materials should not be related to structural phase transition. Notice also in Fig. 1a that when the BNT sample was subjected to reduction in high-vacuum at 1123 K for 6 h, a decrease in the dielectric permittivity is registered, especially towards the temperature region where the peak develops. This led us to consider other characterization techniques to get further insights into the physical mechanism behind the occurrence of this dielectric anomaly, such as mechanical spectroscopy and thermally stimulated depolarization current (TSDC). The results of these characterizations are shown in Fig. 1b,c, respectively, for the as-sintered BNT sample.

Young’s modulus characterization shows two anomalies (labelled as $\text {T}_{R-T}$ and $\text {T}_{T-C}$), which are compatible with the rhombohedral-tetragonal and tetragonal-cubic phase transitions as previously reported^18,19,22^. The internal friction ($Q^{-1}$) measurements showed four anomalies. For temperatures around 500 K, a peak ($\text {T}_{1}$) is observed, which in accordance with discussions presented by Cordero et al.^22^, can be related with a non-trivial transformation between rhombohedral and tetragonal structures over an extremely broad range of temperatures with a residual modulation of the rhombohedral phase, giving rise to antiferroelectric properties until at 563 K, when the modulation disappears and the structure becomes homogeneous. Around 570 K, the sharp internal friction peak is related to the rhombohedral-tetragonal phase transition ($\text {T}_{R-T}$). From 600 to 800 K, $Q^{-1}$ showed a frequency-dependent broad peak ($\text {T}_{2}$). Cordero et al.^22^ reported frequency-dependent processes for $Q^{-1}$, in BNT materials at the kHz region, would be associated with the relaxation of (ferroelastic) domain walls. A detailed analysis of the $Q^{-1}$ behaviour in that work^22^ revealed a small shoulder around 800 K compatible with the $\text {T}_{2}$ anomaly. The height of the $\text {T}_{2}$ peak is frequency-dependent and it disappears at high frequencies; therefore, the physical mechanism related to this peak would be relaxed at the kilohertz frequency range. Another anomaly, for $Q^{-1}$, can be observed around 820 K, which is compatible with the tetragonal to cubic phase transition. The sharp peak, expected in the $Q^{-1}$ for this transformation, is overlapped by the broad peak $\text {T}_{2}$; therefore, a shoulder is observed in that temperature region.

### TSDC modelling

The current data (Fig. 1c) suggest the occurrence of, at least, three principal depolarization processes. Temperature of the peak found at 492 K is compatible with that reported for ferroelectric depolarization ($\text {P}_{F}$) in BNT^19,21,22,30^. In addition, the shoulder observed at 554 K locates towards the temperature region where the rhombohedral (*R*3*c*) to tetragonal (*P*4*bm*) phase transition ($\text {P}_{R-T}$) of BNT manifests^18,21^. Unexpectedly, a strong and broad peak ($\text {P}_{HT}$) develops towards the temperature range between 590 and 795 K where, say again, no phase transition occurs^18^. This peak is one magnitude order higher than that arising from the ferroelectric depolarization ($\text {P}_{F}$). It is known that migration of ionic charge carriers over macroscopic distances may result in high-temperature current peaks usually much more intense than dipolar-related TSDC peaks^52^. Accordingly, the $\text {P}_{HT}$ peak observed in Fig. 1c may have a similar origin, and this is the question disclosed below.

The asymmetrical shape of this huge peak suggests that its incidence involve the overlap of more than one depolarization current processes having similar relaxation times^52^. Multiple TSDC peaks can be described by considering the superposition of individual contributions as^52^:

1 $$\begin{aligned} i(T) = \sum _{n}^{}\Bigg \{\frac{\sigma _{0n}}{\varepsilon \varepsilon _{0}}Q_{0n}\exp {\left( \frac{-E_{n}}{k_{B}T}\right) } \exp {\left[ \frac{-\sigma _{0n}}{\varepsilon \varepsilon _{0} \beta } \int _{T_{0}}^{T} \exp {\left( \frac{-E_{n}}{k_{B}T'}\right) }dT'\right] }\Bigg \} \end{aligned}$$

where $\sigma _{0}$ is the pre-exponential factor of the ionic conductivity, $\varepsilon$ is the relative dielectric permittivity, $\varepsilon _{0}$ the vacuum dielectric permittivity, $\text {T}_{0}$ the initial temperature, $\text {Q}_{0}$ the charge at $\text {T}_{0}$, $\text {E}_{n}$ the activation energy of the $\text {P}_{n}$ process, $\beta$ the heating rate and $\text {k}_{B}$ the Boltzmann constant.

In order to isolate each process contributing to $\text {P}_{HT}$, the thermal cleaning procedure was applied. That is, once the original TSDC spectrum was measured, the next step consisted in taking an equally poled sample and conducting depolarization current measurements towards the temperature range where the first contributing peak is supposed to occur, followed by cooling, and then measurement again of current in that new scenario where the first contributing peak has already been clean (or almost clean). Subtracting these new data to the original curve data leads to peak deconvolution, the procedure of which can be extended for several consecutive overlapped peaks^52^. This procedure allowed us identifying that the broad $\text {P}_{HT}$ peak consists of two processes we named $\text {P}_{1}$ and $\text {P}_{2}$ in Fig. 1c.

In the following, the initial rise method (IRM)^52^ was applied to obtain a first approximation of the activation energies associated with each of these two $\text {P}_{1}$- and $\text {P}_{2}$-related TSDC processes. In this method, for a peak developing (let’s suppose) from $\text {T}_{0}$ to $\text {T}_{1}$, temperatures T approaching $\text {T}_{0}$ should be considered for data processing, so as the second exponential of every contribution in Eq. (1) reduces approximately to 1. This is equivalent to say that each TSDC contribution can be in such a case described by:

2 $$\begin{aligned} i(T) \propto \exp {\left( \frac{-E_{n}}{k_{B}T}\right) } \end{aligned}$$

**Figure 2:**
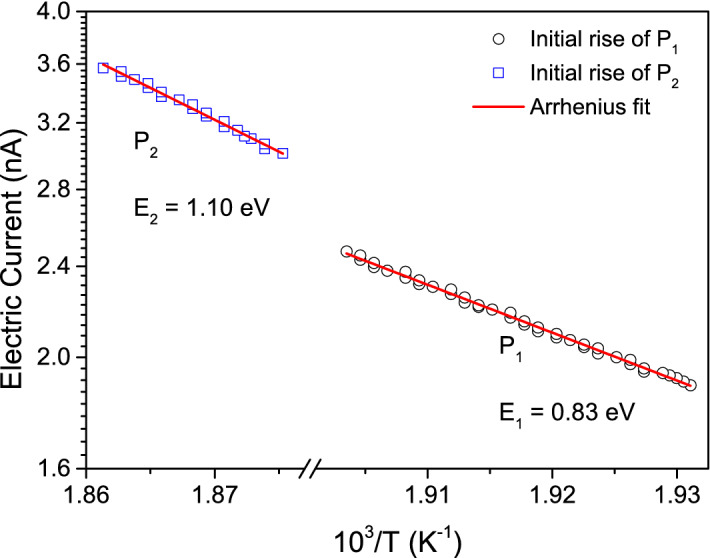
Arrhenius plot of the $\text {P}_{1}$- and $\text {P}_{2}$-related TSDC data considering the initial rise method, ending with fitting using Eq. (2).

Accordingly, an Arrhenius fit of the experimental data measured towards the $\Delta T = (T_{0},T)$ temperature range, with $\text {T} \rightarrow \text {T}_{0}$, allows estimating the activation energy corresponding to the TSDC process under analysis. Figure 2 refers to the results we obtained from application of this method for both the $\text {P}_{1}$- and $\text {P}_{2}$-related TSDC data, and the calculated values of activation energy were 0.83 eV and 1.1 eV, respectively.

Once estimated, these activation energies were used as starting values in Eq. (1) (with $n = 1, 2$) to get the best fit of the whole $\text {P}_{HT}$-related current data in Fig. 1c. For this, values of $\text {E}_{n}$ were initially fixed and the $\sigma _{0n}$ and $\text {Q}_{0n}$ parameters were estimated from the partially best fit. After that, these values of $\sigma _{0n}$ and $\text {Q}_{0n}$ were fixed, and then $\text {E}_{n}$ had its values refined. This procedure was iteratively repeated until getting convergence of all parameters. Final values of $\text {E}_{n}$ , $\sigma _{0n}$ and $\text {Q}_{0n}$ are listed in Table 1.

**Table 1:** Final values of $\text {E}_{n}$, $\sigma _{0n}$ and $\text {Q}_{0n}$ calculated in this work.

	$\text {E}_{n}\,(\text {eV})$	$\sigma _{0n}$ ($\upmu \text {S cm}^{-1}$)	$\text {Q}_{0n}$ ($\upmu \text {C}$)
$n = 1$	0.82	0.0884	2214
$n = 2$	1.11	2.25	4152

During fit, the values of $\varepsilon = 442$ and $\beta = 0.036\,\text {K/s}$ were used.

Values of activation energy were finally 0.82 eV and 1.11 eV for, respectively, the $\text {P}_{1}$- and $\text {P}_{2}$-related TSDC processes. Activation energies in the range 0.6–1.2 eV have been usually attributed to the thermal motion of doubly-ionized oxygen vacancies in perovskite oxides at high temperatures^53–60^. Results in this work reproduce very well the activation energy of 1.11 eV, associated with long-range migration of oxygen vacancies in $\text{K} _{0.5 } \text{Na} _{0.5 } \text{NbO}_{3}$ ceramics^56^ and the values of 0.86 eV and 1.1 eV processed by Liu et al.^57^ for similar TSDC peaks found in Fe-doped $\text {SrTiO}_{3}$ ceramics. Such peaks were associated with relaxation of in-grain oxygen vacancies piled up at grain boundaries and oxygen vacancies across grain boundaries, respectively. It is timely remembering that incidence of such defects is common in perovskite-structured $\text {ABO}_{3}$-based materials sintered at high temperatures^53,61^. In addition, such defects in BNT also arise owing to the trend of some bismuth oxide volatilization towards high temperatures during processing^53,61^. In other words, as proposed by Liu et al. for the parent Fe-doped $\text {SrTiO}_{3}$, incidence of the $\text {P}_{1}$ and $\text {P}_{2}$ TSDC peaks and, thus, prominent $\text {P}_{HT}$ broad peak observed in this work for BNT could also arise from an identical oxygen vacancy-related mechanism.

### Impedance spectroscopy characterizations

**Figure 3:**
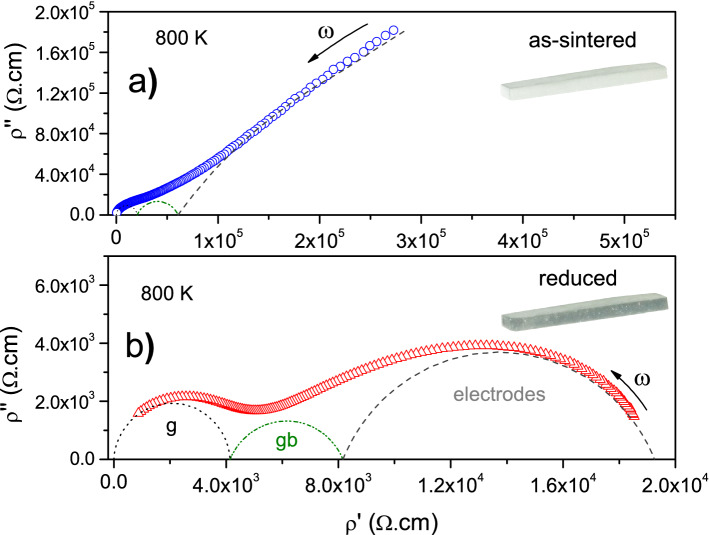
Complex impedance spectra (converted to resistivity), $\rho ''$ versus $\rho '$, measured in (**a**) as-sintered and (**b**) reduced BNT at 800 K. In both figure insets, each BNT sample is shown with its final appearance after sintering or reduction, the latter revealing to be black-like. Semicircles are the deconvolution of the experimental data by using a parallel resistance–capacitance (R-C) network as equivalent circuit. They were attributed to impedance responses arising from the grains (g), grain boundaries (gb), and material-electrode interfaces (electrodes).

To verify this, impedance measurements were performed on as-sintered as well as reduced BNT, and representative results are illustrated in Fig. 3 in terms of imaginary versus real parts of impedance (normalized to resistivity, $\rho$) for the data collected at 800 K. Both spectra consist of three semicircles, each of which can be ideally (Debye model) fitted using a parallel resistance–capacitance (R-C) network as equivalent circuit^62^. Considering a series connection of such networks, the angular frequency ($\omega$) dependence of the total impedance, $Z^{*}(\omega )=Z'(\omega )-jZ''(\omega )$, satisfies:

3 $$\begin{aligned} Z^{*}(\omega ) = \sum _{i}^{} \frac{R_{i}}{1+\omega ^{2} \tau _{ci}^{2}} -j\frac{R_{i}\omega \tau _{ci}}{1+\omega ^{2} \tau _{ci}^{2}} \end{aligned}$$

where *i* denotes the number of semicircles, while $\tau _{ci} \equiv \text {R}_{i}\text {C}_{i}$ refers to the relaxation time involved in the conduction process.

To achieve a better agreement with experimental data, an empirical constant phase element (CPE) parameter was used, in place of the ideal capacitance, to account for relaxation in most solids^62^. One common approach consists in considering the CPE element to have an admittance of the type: $Y_{CPE}(\omega )= Q(j\omega )^{n}$, with $0 \le n \le 1$, different from the ideal capacitance-associated admittance $Y(\omega )=j\omega C$, an expression which is, in the framework of the CPE approach, satisfied when $n = 1$. The parameter *n* has been argued to reveal either the existence of a distribution of relaxation times or a correlation degree of charges interaction (instead of non-interacting dipoles or charges) in real dielectric media^62,63^. All the measured impedance data were fitted using the professional ZView^®^ software^64^, from which *n* was found to range from about 0.87 to 0.99 (i.e., $n \rightarrow 1$), suggesting the Debye model to be in principle a good approximation in this work.

Values of capacitance extracted from the fit scaled in the order of $10^{-10}$ F for the high-frequency semicircle, $10^{-9}$ to $10^{-8}$ F for the intermediate-frequency semicircle and $10^{-7}$ to $10^{-6}$ F for the low-frequency semicircle. Considering a sample geometrical factor of $h/A = 0.02\,\text {mm}^{-1}$ (*A* refers to the electrode area on the sample surface, and *h* the electrodes spacing), these values can be congruently attributed to impedance responses arising from the grains (g), grain boundaries (gb) and material-electrode interfaces (electrodes), from high to low frequencies^62^. We were in the following much concerned with the impedance response from the material, i.e., from grains and grain boundaries in BNT.

The diameter of each semicircle, as in Fig. 3, is known to represent the resistance (resistivity) from each micro-region. Therefore, when comparing between the impedance data from as-sintered versus reduced BNT, it is clear that the last one reveals much more conductive. Electrical conductivity in materials satisfies the relation $\sigma = N q\mu$ , where *N* is the density of charge carries, each with a *q* charge value, while $\mu$ holds for the charge mobility. Accordingly, the huge increase in conductivity in the reduced sample arises from an increase in charge carrier density and/or mobility, as analysed below.

**Figure 4:**
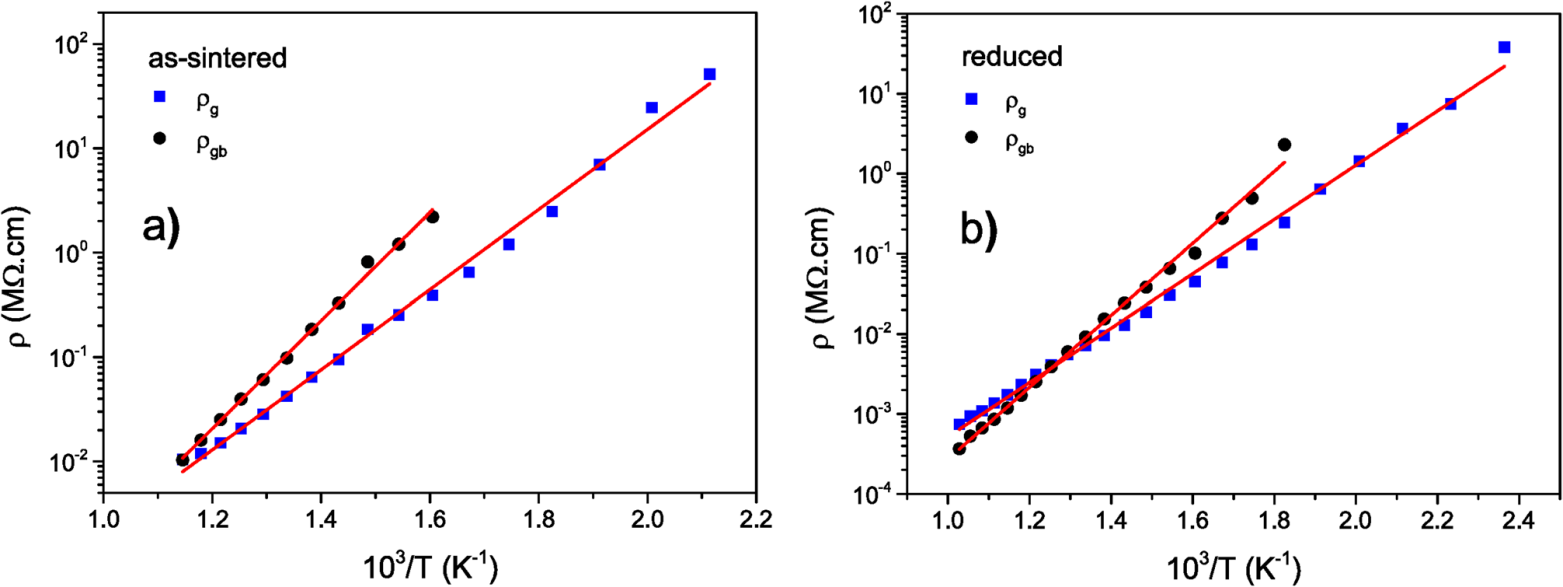
Arrhenius plot for the grain and grain-boundary resistivities from (**a**) as-sintered and (**b**) reduced BNT. The red lines are the linear fit of each set of data using Eq. (4).

Figure 4 depicts the Arrhenius plot for the resistivity from grains and grain-boundaries in as-sintered and reduced BNT. Linear behaviours are observed, suggesting the resistivity to as well obey a relation of the type:

4 $$\begin{aligned} \rho = \rho _{0} \exp {\left( \frac{\Delta E}{k_{B}T}\right) } \end{aligned}$$

where $\rho _{0}$ is the pre-exponential factor and $\Delta E$ the mobility-related activation energy. In as-sintered sample (Fig. 4a), the values of activation energy were $\Delta E_{g}=(0.76 \pm 0.03)$ eV for the grains, and $\Delta E_{gb}=(1.03 \pm 0.05)$ eV for the grain boundaries. These values are characteristic of oxygen vacancies migration in perovskite-structured compounds, as commented above.

When subjected to vacuum at high temperature (reduced BNT), oxygen is removed from the sample, traducing into an increase in oxygen vacancies and, consequently, an increase (decrease) in electrical conductivity (resistivity), as observed in Figs. 3 and 4. The question now is: what about charge carrier mobility in the reduced sample? The values estimated in Fig. 4b for the activation energy were, in this case, $\Delta E_{g}=(0.67 \pm 0.03)$ eV for the grains, and $\Delta E_{gb}=(0.89 \pm 0.04)$ eV for the grain boundaries. These values are sensibly lower than in as-sintered BNT, reflecting then an increase in charge mobility for electrical transport in the reduced sample. This is indicative of a change (at least partially) in the nature of the charge carriers leading the transport process across the material.

A look at the low-frequency semicircle corresponding to the impedance response from the material-electrode interface (Fig. 3) suggests that the electrical conductivity in as-sintered BNT is basically (or mostly) of ionic nature, implying observation (say, trend to occurrence) of a huge semicircle diameter as expected for ion-blocking electrodes. For reduced BNT, in contrast, observation of a significantly-reduced low-frequency semicircle diameter allows concluding on a comparatively semi-blocking effect at identical electrodes. During BNT submission to vacuum, the reaction: $\text {O}^{2-} \rightleftharpoons 1/2\text{O}_{2}(\text {g}) + 2\,\text {e}^{\prime }$ applies, and can be written in its general form as: $\text {O}^{\text{x}}_{\text{O}} \rightleftharpoons 1/2\text{O}_{2}(\text {g}) + \text {V}^{\bullet \bullet }_{\text{O}} + 2\,\text {e}^{\prime }$ to explicitly account for generation of the oxygen vacancies in oxygen-poor atmosphere. Progressively, $\text {Ti}^{4+}$ should reduce to $\text {Ti}^{3+}$ according to the reaction: $\text {Ti}^{4+} + \text {e}^{\prime } \rightleftharpoons \text {Ti}^{3+}$, making the whole process to be fully transcribed as:

5 $$\begin{aligned} \text {O}^{\text{x}}_{\text{O}} + 2\text {Ti}^{\text{x}}_{\text{Ti}} \rightleftharpoons 1/2\text{O}_{2}(\text {g}) + \text {V}^{\bullet \bullet }_{\text{O}} + 2\text {Ti}^{\prime }_{\text{Ti}} \end{aligned}$$

In fact, the BNT sample subjected to vacuum resulted significantly dark, as shown in the Fig. 3b inset, which is the colour trend observed in reduced titanium-containing materials^65,66^. Besides, the whole $\text {Ti}^{\prime }_{\text{Ti}} \equiv \text {Ti}^{3+}_{\text{Ti}}$ defect is known to be mobile through electron hopping between the tri($\text {Ti}^{\prime }_{\text{Ti}} \equiv \text {Ti}^{3+}_{\text{Ti}}$)- and tetra($\text {Ti}^{\text{x}}_{\text{Ti}} \equiv \text {Ti}^{4+}_{\text{Ti}}$)-valent titanium ions, a mechanism to which correspond lower activation energy^67,68^, meaning higher charge mobility when compared to the oxygen vacancy-related mechanism. Reduced BNT is, therefore, to be viewed as a mixed conductor where the total conductivity comprises a parallel connection of ionic and electronic contributions ($\sigma _{total} = \sigma _{ion} +\sigma _{elect}$).

Back to as-sintered BNT, notice that the values of activation energy estimated from the resistivity-related Arrhenius plots illustrated in Fig. 4 are comparable to those values we listed in Table 1, as derived from TSDC through the current-related Arrhenius plot shown in Fig. 2. This further validates the interpretation given above for origin of the $\text {P}_{1}$- and $\text {P}_{2}$-related TSDC processes in Fig. 2, and according to which these are actually related to in-grain and grain-boundary electrical transport responses, respectively.

### Modelling dielectric and mechanical responses at high temperatures

In the framework of the series-layer model applying here for grains and grain-boundaries arrangement in the ceramic material, observation that $\Delta E_{gb} >\Delta E_{g}$ means that the grain boundaries are essentially resistive micro-regions separating conductive grains^62^. The consequence of such a scenario is partial accumulation of charge carriers at the semi-blocking grain boundaries during electrical transport across the material. Just noting that the complex capacitance is linked to the impedance by the following equation: $C^{*}(\omega ) = C'(\omega ) -jC''(\omega )= [j\omega Z^{*}(\omega )]^{-1}$, from which consideration of two parallel R-C networks representing the impedance of, e.g., grains (g) and grain boundaries (gb), i.e., Eq. (3) (with $i = 1, 2$), implies:

6 $$\begin{aligned} C^{*}(\omega ) = \left[ C_{\infty }+\frac{C_{s}-C_{\infty }}{1 +\omega ^{2} \tau _{p}^{2}}\right] -j\left[ \frac{1}{\omega (R_{g}+R_{gb})}+\frac{(C_{s}-C_{\infty })\omega \tau _{p}}{1 +\omega ^{2} \tau _{p}^{2}}\right] \end{aligned}$$

where $C_{\infty }=\tfrac{C_{g}C_{gb}}{C_{g}+C_{gb}}$ is the limiting high-frequency capacitance, $C_{s}=\tfrac{C_{g}R_{g}^{2}+C_{gb}R_{gb}^{2}}{(R_{g}+R_{gb})^{2}}$ (with $C_{s}>C_{\infty }$) is the low-frequency capacitance arising from the interfacial polarization process at grain boundaries, while $\tau _{p}=\tfrac{R_{g}R_{gb}(C_{g}+C_{gb})}{R_{g}+R_{gb}}$ refers to the resulting polarization-related relaxation time. (Just remembering that capacitance and dielectric permittivity are linked through: $C^{*}=\varepsilon _{0}\varepsilon ^{*}\tfrac{A}{h}$; $\varepsilon ^{*}=\varepsilon ' -j\varepsilon ''$.)

**Figure 5:**
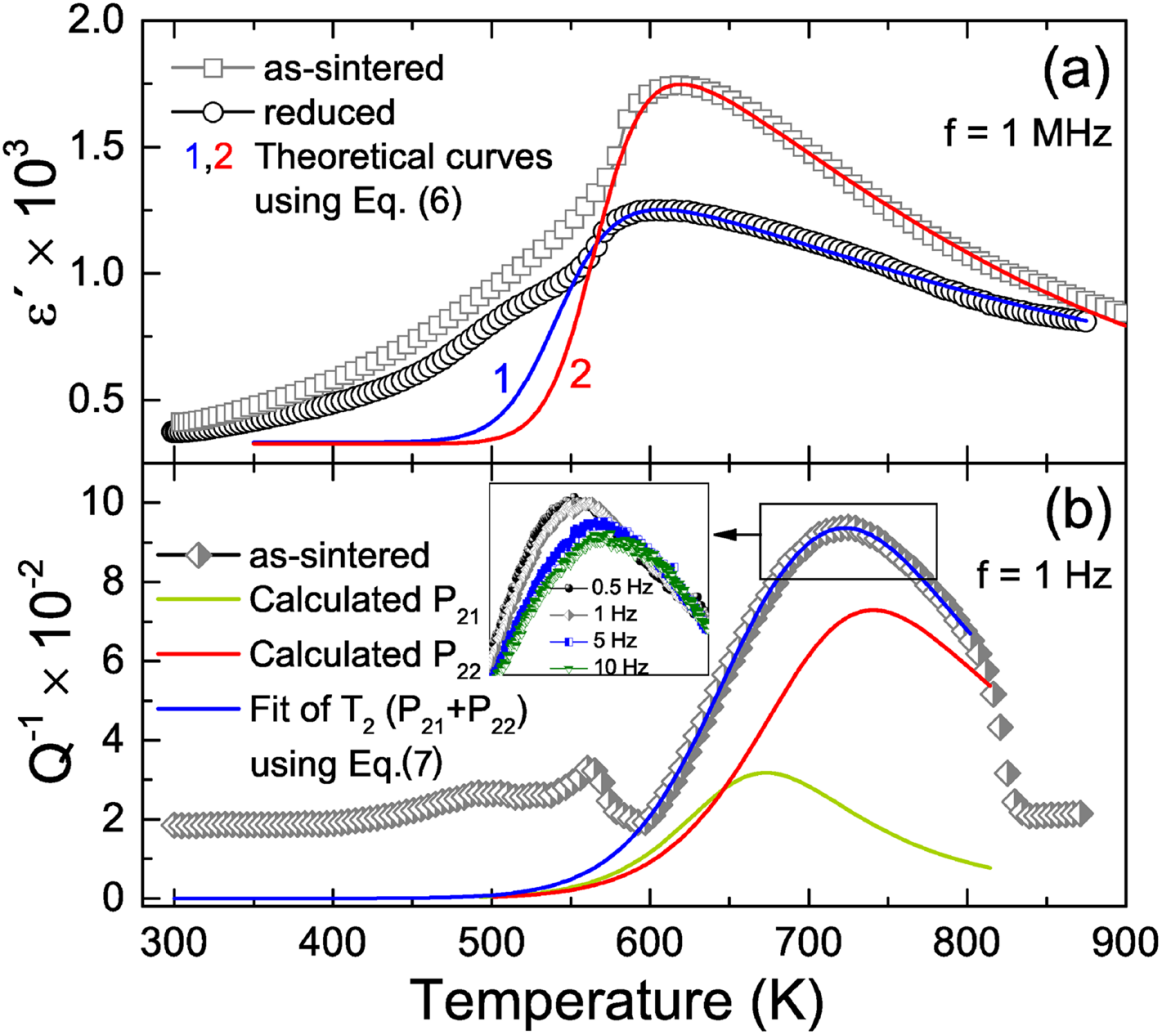
(**a**) Fitting of the experimental dielectric permittivity, at 1 MHz, as a function of temperature for as-sintered and reduced BNT samples, using the real part of the Eq. (6). (**b**) Fitting of the internal friction ($Q^{-1}$) as a function of temperature, obtained at 1 Hz, for as-sintered BNT sample, using Eq. (7) (the best fitting parameters of Eq. (7) are summarized in Table 2). Dispersion around the peak at high temperature is highlighted in the inset.

Equations (3) and (6) have been widely used to corroborate (data fit included, in many cases) the dielectric response observed in varied electroceramics^58,69^. Such data processing procedure was here considered to see whether it is possible to reproduce the dielectric peak observed around 610 K in Fig. 1a, in which case temperature dependence of resistivities and corresponding activation energies were taken into account. Figure 5a shows the thermal behaviour of the real part of dielectric permittivity ($\varepsilon '$) from as-sintered and reduced BNT samples, now together with the theoretical curves then simulated from the real part of Eq. (6) after evaluating the available electrical parameters, that were deduced from the TSDC and impedance spectroscopy measurements (i.e., $R_{0}^{g} = 53.54$ $\text{m}\Omega$, $R_{0}^{gb} = 2.13$ $\text{m}\Omega$, $\Delta E_{g}=0.82$ eV, $\Delta E_{gb} = 1.11$ eV, $C_{g} = 0.20$ nF, $C_{gb} = 1.20$ nF, for as-sintered BNT, and $R_{0}^{g} = 35.0$ $\text{m}\Omega$, $R_{0}^{gb} = 3.80$ $\text{m}\Omega$, $\Delta E_{g} = 0.67$ eV, $\Delta E_{gb} = 0.89$ eV, $C_{g}= 0.16$ nF, $C_{gb} = 0.83$ nF for reduced BNT). These curves reproduce well the experimental data related to this high-temperature dielectric peak.

The same factors that give rise to the high-temperature dielectric peak can also cause the broad $\text {T}_{2}$ peak, of $Q^{-1}$, shown in Fig. 1b between 600 and 800 K. Thus, a similar approach to that used in the dielectric case can be applied to try to reproduce the behaviour of $Q^{-1}$ in this temperature range. The thermally activated nature of this peak together with its broad and asymmetrical shape suggest physical mechanisms with a distribution of relaxation times. One can then consider the Fuoss-Kirkwood approach^70^, which when applied to anelastic processes describes the temperature dependence of $Q^{-1}$ as:

7 $$\begin{aligned} Q^{-1}= & {} \sum _{i}\dfrac{\Delta _{i}}{T\cosh ^{2}\left( \dfrac{a_{i}}{2\kappa _{B}T}\right) }\frac{1}{(\omega \tau _{i})^{\alpha _{i}}+(\omega \tau _{i})^{-\alpha _{i}}} \end{aligned}$$

8 $$\begin{aligned} \tau=\tau _{0}\exp \left( \frac{E}{\kappa _{B}T}\right) {{\,\text{sech}\,}}\left( \frac{a}{2\kappa _{B}T}\right) \end{aligned}$$

where $\tau$ is the relaxation time between two sites with energy separation *a*, $\Delta$ is a constant proportional to the point defect concentration, to the elastic modulus and the change in the distortions because the point defect mobility and $\alpha$ is the Fuoss-Kirkwood width parameter^70^, that being 1 returns the Debye equation with single relaxation time, and values less than 1 implies that the relaxation process involves more than one relaxation time.

Figure 5b shows that a very good fitting of the experimental $Q^{-1}$ data between 600 and 800 K was achieved when two physical processes, $\text {P}_{21}$ and $\text {P}_{22}$, with activation energies of 0.82 and 1.11 eV, respectively, are considered. The best fitting parameters, which are summarized in Table 2, were used to calculate the relaxation time (from Eq. (8)) at the peak temperature for each process and its reciprocal, the relaxation frequency $f_{R}$. The calculated values of $f_{R}$, 4.7 and 0.9 Hz from $\text {P}_{21}$ and $\text {P}_{22}$ processes, respectively, are in accordance with the strong frequency dependence on the height of $\text {T}_{2}$, between 0.1 and 10 Hz, shown in the inset of Fig. 5b. Also, these $f_{R}$ values justify the absence of this $\text {T}_{2}$ peak from $Q^{-1}$ profiles measured at the kHz frequency range^22^. In fact, the viscous behaviour of the complex anelastic response at high temperatures can be directly related to physical processes with long relaxation times, such as $\text {P}_{21}$ and $\text {P}_{22}$, which arise from local stresses generated by the accumulation of ionic space charges. These mechanisms may also be responsible for the unusual presence of ferroelastic domains in BNT in the same temperature range^71^.

**Table 2:** Best fitting parameters of the $\text {P}_{21}$ and $\text {P}_{22}$ peaks in BNT anelastic response at high temperatures processes, according to Eqs. (7) and (8).

	${\tau _{0}}$ (s)	E (eV)	*a* (eV)	${\alpha }$	${\tau }$ (s)	${f}_{R}$ (Hz)
$\text {P}_{21}$	$2.21\times 10^{-7}$	0.82	0.170	0.952	0.211	4.7
$\text {P}_{22}$	$1.08\times 10^{-8}$	1.11	0.257	0.655	1.17	0.9

The results are outstanding, not only the high-temperature dielectric behaviour, but also the TSDC profile and the mechanical response of BNT have been successfully reproduced using well-established physical models, which consider the BNT constituted by conductive grains and semi-blocking grain boundaries, with the accumulation of oxygen vacancies at grain boundaries, and consequently, with interfacial polarization.

## Conclusions

In summary, dense BNT bodies were prepared through the conventional ceramic method and then studied in terms of thermal behaviour of dielectric permittivity. As has been reported elsewhere, a prominent and broad dielectric peak was found at high temperatures. Its origin was assessed by applying two different approaches, namely, depolarization current that revealed as well occurrence of a huge and broad peak towards identical temperature range, resulting from the overlap of two ionic processes identified as arising from bulk and grain boundaries, and impedance spectroscopy from which this association was also verified. Conducting permittivity and impedance measurements not only on as-sintered but also reduced BNT allowed concluding that the electrical transport process in this material is originally led by oxygen vacancies. In terms of model, all the depicted scenario was that of a material consisting of conductive grains and semi-blocking grain boundaries, causing the development of interfacial polarization at grain-to-grain contacts. Through simulation of dielectric permittivity and internal friction by considering theoretical equations deduced in accordance to this model and the parameters inferred from the conducted experiments, this work has demonstrated that this interfacial mechanism is actually responsible not only for occurrence of the broad high-temperature dielectric permittivity response of concern in this report but also for the development of an unusual internal friction peak at high temperatures and low frequencies, which may also be responsible for the presence of ferroelastic domains in BNT at high temperatures.

## Methods

### Sample preparation

$\text {Bi}_{0.5}\text {Na}_{0.5}\text {TiO}_{3}$ (BNT) was synthesized by solid-state reaction using high-purity $\text {Bi}_{2}\text {O}_{3}$ (Acros Organics, 99.9 %), $\text {Na}_{2}\text {CO}_{3}$ (Acros Organics, 99.8 %) and $\text {TiO}_{2}$ (Sigma-Aldrich, 99.8 %) reagents, which were weighed according to the nominal composition, and then mixed in a ball mill for 24 h. This powder was calcined at 1173 K for 3 h, and ball milled again for 24 h to reduce particle sizes. Finally, this powder was uniaxially pressed at 150 MPa into cylindrical- or plate-like thick samples, followed by isostatic pressing at 250 MPa, and then sintering at 1393 K for 3 h.

### Apparent density and structural characterizations

Apparent density was measured by Archimedes method, using a precision balance (AUW220D, Shimadzu) and distilled water as medium. Relative density was estimated as 98 % of BNT theoretical density ($\text {TD} = 5.99\,\text {g/cm}^{3}$)^18^. X-ray diffraction (XRD) data from this material, collected using a LabX XRD-6000 Shimadzu diffractometer, allowed verifying that its room temperature (RT) crystallographic structure matches the *R*3*c* symmetry (ICSD Code 280983 card), as expected^18^.

### Electrical characterizations

Platinum electrodes were sputtered on both major surfaces of the disk- or plate-like shaped samples and, then, dielectric permittivity and complex impedance were separately measured from RT to 970 K using an IET 7600 Plus high-precision LCR meter, over the 10 Hz to 1 MHz frequency range. These measurements were conducted on as-sintered BNT as well as sintered BNT subjected to reduction in vacuum ($1 \times 10^{-7}$ Torr) at 1123 K for 6 h, which was labelled as reduced BNT. A set of BNT samples were poled at 330 K during 40 min under an electric field of 4 kV/mm, followed by polarization frozen to RT. Then, thermally stimulated depolarization current (TSDC) was recorded from RT to 870 K, under a heating rate of about 2 K/min, using a picoammeter Keithley model 6485. The data were processed following well established procedures, including the thermal cleaning protocol when facing overlapped contributions from different processes with similar relaxation times^52^.

### Mechanical spectrocopy characterizations

The complex Young’s modulus ($M^{*}=M^{'}+iM{''}$) of the as-sintered BNT samples was measured using a Dynamic Mechanical Analyzer (DMA), PerkinElmer model DMA8000, in a three-point bending geometry, at frequencies between 0.1 and 20 Hz, and temperatures from RT to 870 K, with heating and cooling rates of 1 K/min, in air. The mechanical spectra are displayed in terms of the real part of the complex Young’s modulus ($M^{'}$) and the elastic energy loss coefficient $Q^{-1}=M{''}/M{'}$^70^.
